# Inactivation of *bpsl1039-1040* ATP-binding cassette transporter reduces intracellular survival in macrophages, biofilm formation and virulence in the murine model of *Burkholderia pseudomallei* infection

**DOI:** 10.1371/journal.pone.0196202

**Published:** 2018-05-17

**Authors:** Peechanika Pinweha, Pornpan Pumirat, Jon Cuccui, Niramol Jitprasutwit, Veerachat Muangsombut, Varintip Srinon, Usa Boonyuen, Parameth Thiennimitr, Paiboon Vattanaviboon, Felipe Cia, Sam Willcocks, Gregory J. Bancroft, Brendan W. Wren, Sunee Korbsrisate

**Affiliations:** 1 Department of Immunology, Faculty of Medicine Siriraj Hospital, Mahidol University, Bangkok, Thailand; 2 Department of Microbiology and Immunology, Faculty of Tropical Medicine, Mahidol University, Bangkok, Thailand; 3 Faculty of Infectious and Tropical Diseases, London School of Hygiene and Tropical Medicine, London, United Kingdom; 4 Department of Molecular Tropical Medicine and Genetics, Faculty of Tropical Medicine, Mahidol University, Bangkok, Thailand; 5 Department of Microbiology, Faculty of Medicine, Chiang Mai University, Chiang Mai, Thailand; 6 Laboratory of Biotechnology, Chulabhorn Research Institute, Bangkok, Thailand; East Carolina University Brody School of Medicine, UNITED STATES

## Abstract

*Burkholderia pseudomallei*, a gram-negative intracellular bacillus, is the causative agent of a tropical infectious disease called melioidosis. Bacterial ATP-binding cassette (ABC) transporters import and export a variety of molecules across bacterial cell membranes. At present, their significance in *B*. *pseudomallei* pathogenesis is poorly understood. We report here characterization of the BPSL1039-1040 ABC transporter. *B*. *pseudomallei* cultured in M9 medium supplemented with nitrate, demonstrated that BPSL1039-1040 is involved in nitrate transport for *B*. *pseudomallei* growth under anaerobic, but not aerobic conditions, suggesting that BPSL1039-1040 is functional under reduced oxygen tension. In addition, a nitrate reduction assay supported the function of BPSL1039-1040 as nitrate importer. A *bpsl1039-1040* deficient mutant showed reduced biofilm formation as compared with the wild-type strain (*P* = 0.027) when cultured in LB medium supplemented with nitrate under anaerobic growth conditions. This reduction was not noticeable under aerobic conditions. This suggests that a gradient in oxygen levels could regulate the function of BPSL1039-1040 in *B*. *pseudomallei* nitrate metabolism. Furthermore, the *B*. *pseudomallei bpsl1039-1040* mutant had a pronounced effect on plaque formation (*P* < 0.001), and was defective in intracellular survival in both non-phagocytic (HeLa) and phagocytic (J774A.1 macrophage) cells, suggesting reduced virulence in the mutant strain. The *bpsl1039-1040* mutant was found to be attenuated in a BALB/c mouse intranasal infection model. Complementation of the *bpsl1039-1040* deficient mutant with the plasmid-borne *bpsl1039* gene could restore the phenotypes observed. We propose that the ability to acquire nitrate for survival under anaerobic conditions may, at least in part, be important for intracellular survival and has a contributory role in the pathogenesis of *B*. *pseudomallei*.

## Introduction

*Burkholderia pseudomallei* is a non-spore-forming, gram-negative bacillus that causes a severe, human, tropical infectious disease called melioidosis [1]. This saprophytic and facultative intracellular bacterium is found in the soil and water within endemic areas, including Southeast Asia and Northern Australia. Recently, Limmathurotsakul et al. [2] estimated that there are 165,000 human melioidosis cases worldwide annually, from which 89,000 people die, demonstrating the underappreciated importance of this severe infectious disease. In Thailand, where melioidosis is endemic, the annual incidence in 2006 was 21.3 per 100,000 people [3]. Melioidosis is acquired through cutaneous inoculation, inhalation and aspiration. The most severe manifestation of the disease is septic shock, which is often associated with acute pneumonia [4]. Therapeutic treatment is difficult even with improvements in diagnosis and antibiotic regimens. Treatment with ineffective antimicrobials may result in case fatality rates exceeding 70% [2]. At present, there is no licensed vaccine against *B*. *pseudomallei*. There is a need to develop a deeper understanding of *B*. *pseudomallei* pathogenesis and to identify potential vaccines to prevent this life-threatening bacterial disease.

Bacterial ATP-binding cassette (ABC) transporters function as versatile systems for the import and export of a variety of molecules across cell membranes [5]. The ABC transporter protein family is one of the largest gene families in most organisms. Generally, ABC transporters are composed of two functional partners: i) an integral transmembrane protein, which forms a channel for substrates to be transported; and ii) a cytoplasmic ATP-binding protein (ATPase), which binds to and hydrolyzes ATP to enable substrate translocation. In bacteria, acquisition of essential nutrients from the host is a pre-requisite for replication and hence to cause successful infection. Bacterial ABC transporters have been reported to be involved in the uptake of nutrients and trace elements, but also the secretion of extracellular toxins, the extrusion of noxious substances and the conferring of multidrug resistance [6]. The significance of ABC transporters in bacterial pathogenesis has been reported in both gram-positive and gram-negative bacteria, and they often play an important role in intracellular bacterial pathogens [7, 8].

Genome sequence analysis has identified approximately 340 genes that encode potential ABC systems across the two chromosomes of *B*. *pseudomallei* [9]. Cuccui et al. [10] used signature-tagged transposon mutagenesis (STM) in *B*. *pseudomallei* and screened the library in a BALB/c mouse intranasal model of infection. A *B*. *pseudomallei* attenuated mutant with a transposon (*Tn*) insertion in *bpsl1039* (encoding a putative transmembrane component of an ABC transporter) was identified. However, the underlying mechanism of the *bpsl1039* attenuation was never elucidated.

In this study, biological assays were used to identify the function of, and the substances transported by, the BPSL1039-1040 ABC transporter. Phenotypes of the *bpsl1039 Tn* insertion (6H4) mutant were characterized, including survival in phagocytic and non-phagocytic cells, biofilm formation and the ability to infect a mouse model, to explore the relative importance of this ABC transporter system in the virulence of *B*. *pseudomallei* infections.

## Materials and methods

### Bacterial strains, growth conditions, and cell lines

*Escherichia coli* DH5 alpha [11], *E*. *coli* RHO3 [12] and *B*. *pseudomallei* (wild-type K96243, 6H4 mutant, 6H4/pME1039) strains were routinely grown in Luria-Bertani (LB) broth or agar (Hardy Diagnostics, Santa Maria, CA) at 37°C. Antibiotics (namely, 600 μg/mL kanamycin or 60 μg/mL tetracycline) were added to the culture medium if required. HeLa (human cervical carcinoma) and J774A.1 (murine macrophage-like cells) were obtained from the American Type Culture Collection (ATCC) and routinely maintained in RPMI 1640 (GIBCO, Invitrogen, Carlsbad, CA) or Dulbecco’s Modified Eagle medium (DMEM; GIBCO, Invitrogen, Carlsbad, CA), supplemented with 10% heat-inactivated fetal bovine serum (HyClone, South Logan, UT) and grown under a 5% CO_2_ atmosphere at 37°C in a humidified incubator. Reagents were purchased from Sigma—Aldrich (St. Louis, MO), unless indicated otherwise.

### Reverse Transcriptase (RT)-PCR analysis

To observe the expression of *bpsl1039-1040*, total RNA from log phase growth of *B*. *pseudomallei* were extracted by InviTrap Spin Universal RNA Mini Kit (Stratec Molecular, Berlin, Germany) according to the manufacturer’s protocol before cDNA synthesis using SuperScript III one step RT-PCR system (Invitrogen, Carlsbad, CA, USA). Amplifications of *bpsl1040* cDNA were performed using primers 1040-F (5’AAAACCCGAATGCTGTC AA3’) and 1040-R (5’TTGAACGGCACCTTGATCT3’) whereas *bpsl1039-bpsl1040* cDNA were amplified with primers 1039-F2 (5’GACGTTCAGCTTCTACCAGTCGCGCCTAT CCGAACGACTATC3’) and 1040-R (5’TTGAACGGCACCTTGATCT3’). The amplification was performed with the following cycling conditions: 95°C (40 sec), 55°C (40 sec) and 72°C (40 sec or 75 sec) for 35 cycles. Amplification of *B*. *pseudomallei* 16S ribosomal RNA was included as a control, using primers 16S-F (5’AGACACGGCCCAGAC TCCTAC3’) and 16S-R (5’CAGTCACCAATGCAGTTCCCA3’).

### Structural modeling of BPSL1039 ABC transporter membrane protein and docking

Initially, the amino-acid sequence of the *B*. *pseudomallei* BPSL1039 ABC transporter membrane protein was blasted against the Protein Data Bank (PDB) database to identify possible templates for homology modeling (http://blast.ncbi.nlm.nih.gov/Blast.cgi). However, no possible template was identified; therefore, the sequence was submitted to the Phyre^2^ server (http://www.sbg.bio.ic.ac.uk/~phyre2/html/page.cgi?id=index) for fold-recognition modeling [13]. The model was constructed using the intensive mode, in which all parameters were set to the default values. In brief, alignments were carried out using hidden Markov models and HHsearch [14]. Two protein structures, PDB ID: 2ONK and 2R6G, were selected as templates based on heuristics to construct the model. *Ab initio* folding simulation (Poing^2^) was also used to model three regions covering 69 residues in the protein (region 1: residues 1–22, region 2: residues 346–372 and region 3: residues 567–585) when no detectable homology to known structures was found [15]. The structural model was verified by Procheck [16], and the docking was analysed by CLC Drug Discovery Workbench.

### Complementation of 6H4 transposon insertion mutant

In order to complement the *B*. *pseudomallei* 6H4 mutant, a 2,047-bp complete ORF of *B*. *pseudomallei bpsl1039* gene was amplified with primers 1039-F3 (5’TGAATTCTCG TCGTCCGCAAGGTT3’) and 1039-R3 (5’AGGTACCGCTCTCGG TCGGTCTCAAT3’), cloned in-frame into pME6032 shuttle vector [17], generating the recombinant plasmid designated pME1039. The recombinant plasmid was delivered into the *B*. *pseudomallei* 6H4 mutant by electroporation (Bio-Rad Laboratories) using the electroporation parameters of 2.5kV, 200Ω and 25μF.

### Nitrate acquisition and *B*. *pseudomallei* growth under anaerobic condition

Overnight cultures of *B*. *pseudomallei* wild-type, 6H4 mutant and the complemented strains were washed and subcultured at a concentration of 1×10^7^ colony forming units (CFU)/mL in M9 medium supplemented with 0.1% casamino acids and 40 mM sodium nitrate (NaNO_3_). The latter was included as a major source of nitrogen. The bacterial culture was incubated statically at 37°C under aerobic or anaerobic atmospheres for comparison purposes. Anaerobic growth studies were performed within an anaerobic jar using an Oxoid AnaeroGen sachet (Thermo Scientific, MA, USA). *B*. *pseudomallei* growth at various time points was observed by the optical density at 600 nm (OD_600_). The measurement and detection of viable bacteria was observed by plating serial dilutions of bacterial samples on LB agar and colony counts.

### Nitrate reduction assay

Nitrate reductase activity was measured by determining the production of nitrite in intact cells with modifications [18]. Essentially, *B*. *pseudomallei* wild-type, 6H4 mutant and the complemented strains were grown statically in LB broth containing 40 mM NaNO_3_ at 37°C for 3 h for the induction of nitrate reductase expression. Cells were suspended in 1× phosphate-buffered saline (PBS) and stored on ice. To test unpermeabilised cells, 0.1 mL of each bacterial suspension was diluted in 0.7 mL of 1×PBS before the assay. To test permeabilised cells, the bacterial membrane was destroyed by adding 20 μL of 0.1% SDS and 40 μL of chloroform to 0.8 mL of the bacterial suspension. The suspensions of the permeabilised or unpermeabilised cells were mixed with 0.1 mL of a solution containing 0.5 mg/mL of methyl viologen. Reactions were initiated by adding 0.1 mL of a solution containing 8 mg/mL of Na_2_S_2_O_4_, 8 mg/mL of NaHCO_3_, and 0.5 M NaNO_3_. Reactions were terminated by vigorous vortex mixing (to oxidize the viologen) and the addition of 1 mL each of sulfanilic acid and *N*-1-naphthylethylenediamine solutions. The nitrate reductase activities of the permeabilised and unpermeabilised bacterial cells were expressed in arbitrary units and were calculated from the formula units, as described previously [18].

### Biofilm formation assay

Analysis of the *B*. *pseudomallei* biofilm formation was performed by a microtiter-plate assay under aerobic and anaerobic conditions in LB broth according to Andrea et al., [19] with some modifications. The bacterial cultures in LB broth with or without 40 mM sodium nitrate supplementation were standardized to a concentration of 1×10^8^ CFU/mL, inoculated into 96-well plates, and incubated 3 days under aerobic or anaerobic condition. The non-adherent bacteria were then carefully removed, and the biofilm was washed twice with sterile 1×PBS and then fixed with methanol for 15 min. The biofilm was subsequently stained with 0.1% crystal violet, and the dye bound to the adherent cells was solubilised with 33% (v/v) glacial acetic acid. Each *B*. *pseudomallei* strain was assayed in at least eight replicates, along with negative controls. The optical density was measured at 630 nm using a microplate reader (Bio-Rad). The biofilm formation capacity was calculated as the absorbance of the test strain divided by the absorbance of the negative control.

### Plaque formation, bacterial invasion and intracellular survival assays

A plaque formation assay [20] was performed using HeLa cells as described earlier, but with some modifications. Briefly, the cell monolayers were infected with *B*. *pseudomallei* at a multiple of infection (MOI) of 20, and then incubated for 2 h to allow bacterial entry before being overlaid with a medium containing gentamicin (128 μg/mL) and spectinomycin (256 μg/mL) to kill extracellular bacteria. The infected monolayers were washed with 1xPBS and stained with 10% crystal violet at 24 h post-infection (p.i.). Plaques were enumerated under an inverted microscope.

Bacterial invasion assays were performed using a HeLa cell line as previously described [20]. Cell monolayers were infected with *B*. *pseudomallei* and incubated for 1 h. The infected monolayers were washed with prewarmed 1xPBS before adding fresh culture medium containing gentamicin and spectinomycin. For quantitation of the bacterial invasion, the infected monolayers were lysed with 0.1% Triton X-100 at 2 h p.i. and plated on trypticase soy agar (TSA).

For the intracellular survival assay [20], HeLa or J774A.1 cell monolayers were infected with *B*. *pseudomallei* at MOI of 1 or 0.1, for 2 h. After infection, the monolayers were washed and overlaid with a medium containing gentamicin (128 μg/mL) and spectinomycin (256 μg/mL). The number of viable intracellular bacteria at indicated time points was quantitated by lysing the host cells with Triton X-100 and the bacteria plated on TSA.

### Mouse infections

Female BALB/c mice (Charles Rivers Laboratories International Inc., UK) aged between 6–8 weeks old were used. The mice were housed under specific pathogen-free conditions, with free access to food and water and environmental enrichment (including Nestlets), and randomly allocated to experimental groups. For infections, 5 mice from each group were infected within a Class I microbiological safety cabinet with aliquots of either *B*. *pseudomallei* wild-type, 6H4 mutant or the complemented strains *via* the intranasal route. A sample of the inoculum was diluted appropriately, plated out on TSA and incubated overnight at 37°C to confirm the actual inoculation dose. For each infection, the mice were anaesthetised intraperitoneally with a combination of Ketamine (50 mg/kg; Ketalar, Pfizer Ltd., UK) and Xylazine (10 mg/kg; Rompun; UK) diluted in pyrogen-free saline and administered in a final volume of 10 ml/kg. Once the mice were anaesthetised, the inoculum was administered by slowly pipetting a total of 50 μL into both nostrils. The mice were then held upright for 30 sec to ensure the liquid had passed into the lungs and were monitored until they had fully recovered from the anaesthetic. Experiments typically lasted 14–30 days. The mice were routinely checked twice daily for signs of illness. If it was determined that they were likely to reach the humane endpoint specified in the Project Licence before the next monitoring event (weight loss exceeding 20% plus signs of clinical illness scored numerically, including an assessment of piloerection, hunching, movement, respiratory pattern, and grimace scale), the mice were immediately culled by cervical dislocation.

### Ethics statement

The *in vivo* experiments of this study, including the sacrifice of mice, were approved by the LSHTM Animal Welfare and Ethical Review Board and performed in accordance with the Animals (Scientific Procedures) Act of 1986 under project licence PPL70/8043 under animal biohazard Containment Level 3 conditions. In data presented, 25 animals were used, of which 10 were culled at end of experiment and 15 culled in anticipation of reaching the humane end point. Euthanasia was cause of death for all animals. None found dead. All the animal care workers have over 15 years of experience working with laboratory animals including *B*. *pseudomallei*-infected mice. They also hold qualifications as NACWO or NTCO (S1 Table).

### Statistical analysis

All *in vitro* assays were conducted at least in triplicate, and an unpaired *t*-test of independent experiments was performed for statistical analysis. The *in vivo* assays were analysed by log-rank tests of survival data, performed using the GraphPad Prism software, version 5.01 (GraphPad Software, San Diego, CA, USA). Results were considered significant at a *P* value of < 0.05.

## Results and discussion

### *B*. *pseudomallei bpsl1039* is co-transcribed with *bpsl1040*, and 6H4 mutant has mutation only at *bpsl1039/bpsl1040* loci

**Fig 1:**
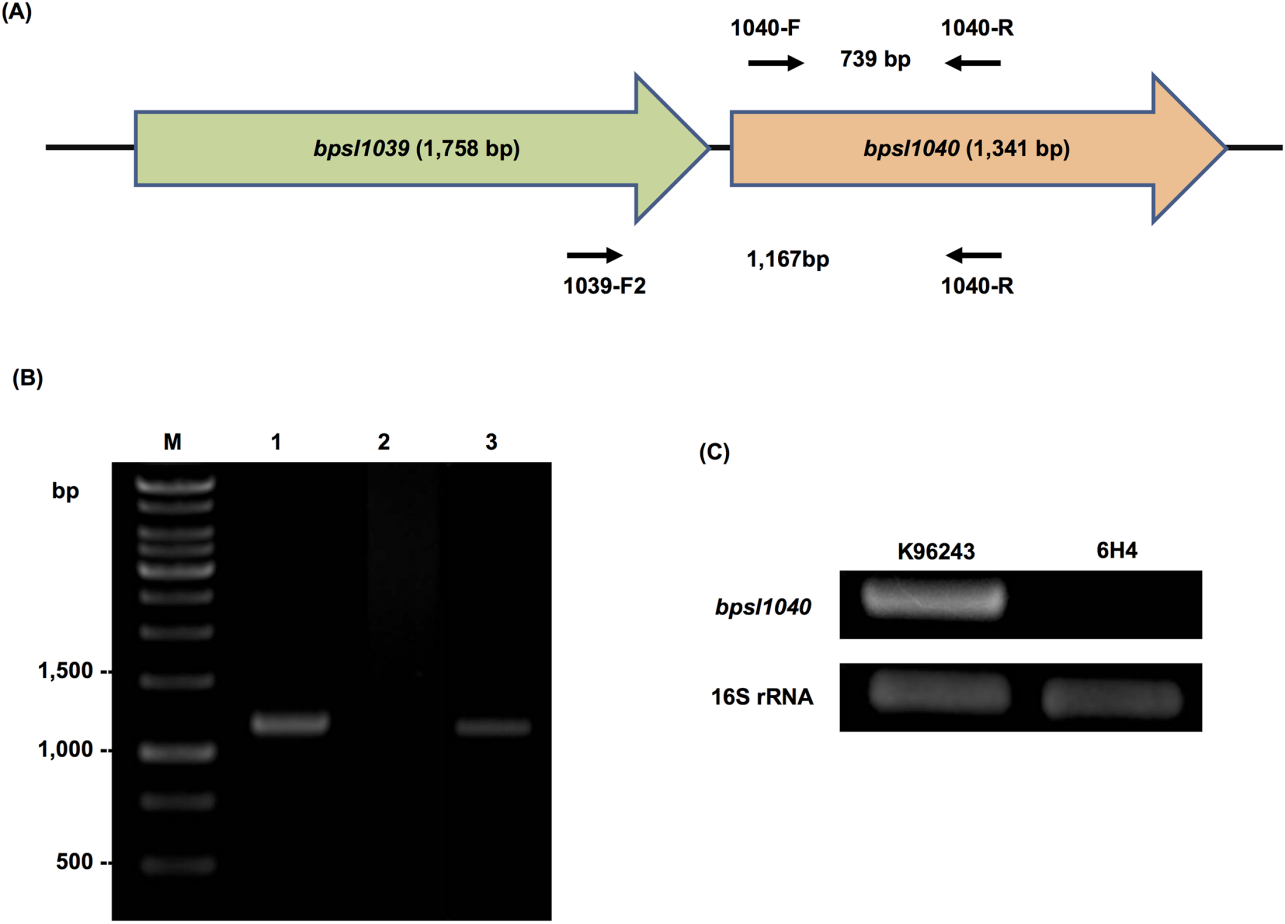
Gene co-localization of *B*. *pseudomallei bpsl1039-bpsl1040* and RT-PCR analysis. (A) Organization of *B*. *pseudomallei bpsl1039-bpsl1040* genes and the location of primer pairs used in RT-PCR analysis. (B) RT-PCR analysis using primers 1039-F2 and 1040-R showed co-transcription of *B*. *pseudomallei bpsl1039-bpsl1040* genes (lane 3). Lanes 1 and 2 represent positive and negative controls, respectively, using wild-type genomic DNA and the extracted RNA, respectively. A negative RT-PCR control (lane 2) confirms that the band observed in the positive reaction is not DNA contamination. (C) RT-PCR analysis of *bpsl1040* expression, using primers 1040-F and 1040-R, was performed in *B*. *pseudomallei* wild-type (K96243) and 6H4 mutant. The 6H4 mutant showed the absence of *bpsl1040* expression. *B*. *pseudomallei* 16S rRNA gene was amplified as control. Lane M represents 1 Kb DNA marker ladder.

*B*. *pseudomallei* K96243 genome analysis [21] revealed that *bpsl1039* is located upstream of *bpsl1040* on chromosome 1 (Fig 1A). RT-PCR analysis of this locus determined that both genes were part of the same transcriptional unit. As shown in Fig 1B, RT-PCR analysis of *B*. *pseudomallei* wild-type cDNA (lane 3), using primers (1039-F2 and 1040-R) spanning *bpsl1039* and *bpsl1040*, amplified a fragment of 1,167-bp in length, indicating that these genes are part of the same transcript. A negative RT-PCR control (Fig 1B, lane 2) confirmed that the band observed in the positive reaction is not DNA contamination. This result is similar to other bacteria [22], in which ABC transporter genes are often clustered in an operon.

In addition, using primers specific for *bpsl1040* only (1040-F and 1040-R), we found a lack of *bpsl1040* transcript in the *B*. *pseudomallei* 6H4 mutant (Fig 1C). In the wild-type K96243 strain cDNA, a 739-bp DNA fragment corresponding to *bpsl1040* DNA was detected (Fig 1C). This strongly suggests that the transposon insertion in the *B*. *pseudomallei* 6H4 mutant caused a deleterious polar effect on *bpsl1040* expression. The upstream gene of *bpsl1039* and downstream gene of *bpsl1040* are annotated to be transcribed in an opposite direction, suggesting that only *bpsl1039* and *bpsl1040* are expressed in the same operon. Taken together, this RT-PCR result suggests that the *B*. *pseudomallei* 6H4 mutant has a mutation in *bpsl1039* and *bpsl1040*.

The *Tn* insertion in *bpsl1039* was previously confirmed by arbitrary PCR using one primer specific for the *Tn* sequence and the other, a degenerate and a nested primer sequencing of the amplified PCR product [10]. This success sequencing suggested that there was no *Tn* insertion at any other locus on the *B*. *pseudomallei* 6H4 strain. Throughout the original STM study [10] and initial analysis during assembly of our library of 1 million *B*. *pseudomallei Tn* mutants [23], we never observed insertion more than once supporting that 6H4 mutant has *Tn* insertion only at the *bpsl1039* and *bpsl1040* loci. However, the possibility of a second *Tn* insertion cannot be ruled out.

### BPSL1039-1040 function as a nitrate importer

Harland et al. [9] predicted the function of BPSL1039-1040 as a nitrate transporter that belonged to a member of the OTCN (Osmoprotectants, Taurine, Cyanate and Nitrate) transporter family. The function of *B*. *pseudomallei* BPSL1039, an integral transmembrane protein, is to form a channel for substrates to be transported. By comparison, the function of BPSL1040, with the signature amino acid motif LSGGQ/R/KQR (at the residues 156–165), is to hydrolyze ATP, enabling substrate translocation. We carried out an amino acid sequence analysis and found that BPSL1039 and BPSL1040 are highly conserved; they shared a 99%–100% (E value = 0) amino acid sequence identity among *B*. *pseudomallei*, *Burkholderia mallei* and *Burkholderia thailandensis* (data not shown).

A docking model was assembled to demonstrate binding of nitrate to BPSL1039. The structural model of the BPSL1039 ABC transporter was shown in S1 Fig. Nitrate and tryptophan were docked into the structural model of BPSL1039ABC transporter using CLC Drug Discovery Workbench. The result showed that only nitrate could be docked into the protein-binding site with a score of minus 12.09, whereas docking tryptophan into the protein was unsuccessful. This result correlates with Harland et al. [9] and our data that *B*. *pseudomallei* BPSL1039-1040 is, at least, a nitrate-binding ABC transporter. Tryptophan was included in the study because it was not predicted to be transported by the BPSL1039-1040 ABC transporter [9].

**Fig 2:**
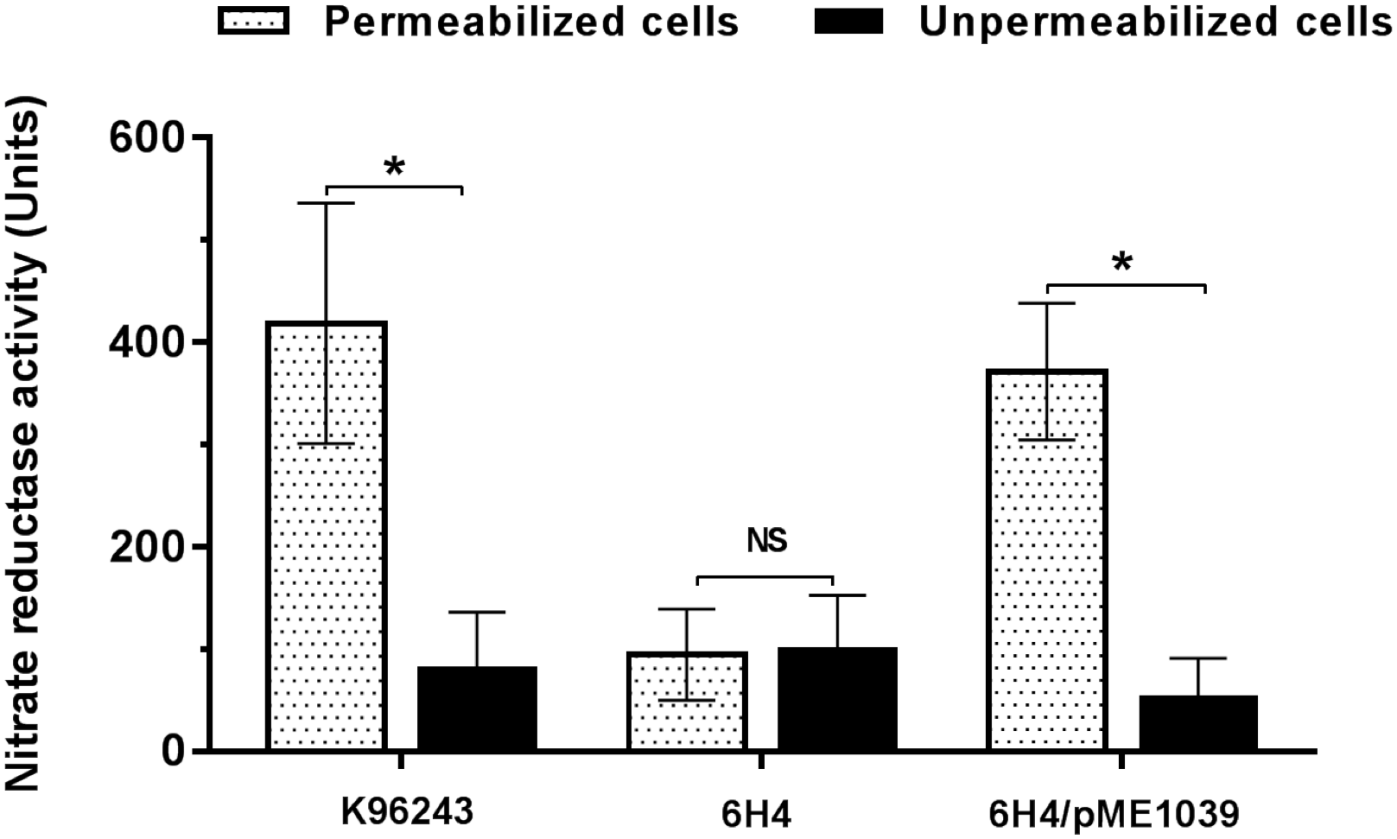
Nitrate reductase activity of *B*. *pseudomallei* wild-type and its derivative strains. *B*. *pseudomallei* K96243 wild-type, 6H4 mutant and 6H4/pME1039 complemented strains were cultured in LB medium supplemented with 40 mM sodium nitrate. The nitrate reductase activitiy of each *B*. *pseudomallei* strain was determined under permeabilised (dot bar) or unpermeabilised conditions (black bar). The level of nitrate reductase activity was measured at absorbance 420 nm and 540 nm. Results are presented as standard errors of the means for experiments done in quadruplicate with two independent experiments. Asterisks indicate significant differences (*P* < 0.05, *t*-test).

Anaerobic growth by bacteria, including *B*. *pseudomallei*, is accomplished through a denitrification enzymes pathway that catalyse the sequential reduction of nitrate to nitrogen gas [24, 25]. Measurement of the nitrate reduction to nitrite in unpermeabilised and permeabilised cell suspensions of the *B*. *pseudomallei* wild-type 6H4 mutant and the *bpsl1039* complemented strains were undertaken to identify the role of BPSL1039/BPSL1040. As shown in Fig 2, permeabilised *B*. *pseudomallei* wild-type K96243 showed a significantly higher level of nitrate reductase activity than their own unpermeabilised cells. In contrast, a significant decrease in activity was observed for the 6H4 mutant compared with the wild-type strain, suggesting a deficiency of the mutant in nitrate transportation from the extracellular space and into the cytosol. As expected, there is no significant difference in enzyme activity between permeabilised and unpermeabilised cells in the 6H4 mutant due to inactivation of the nitrate transporter (Fig 2). Taken together, these data suggest that BPSL1039 acts as a nitrate transporter, allowing extracellular nitrate to react with periplasmic nitrate reductase and yield nitrite. The activity was restored when complementation was carried out with plasmid-borne *bpsl1039*.

### BPSL1039/BPSL1040 ABC transporter enhances anaerobic growth of *B*. *pseudomallei* through nitrate respiration

**Fig 3:**
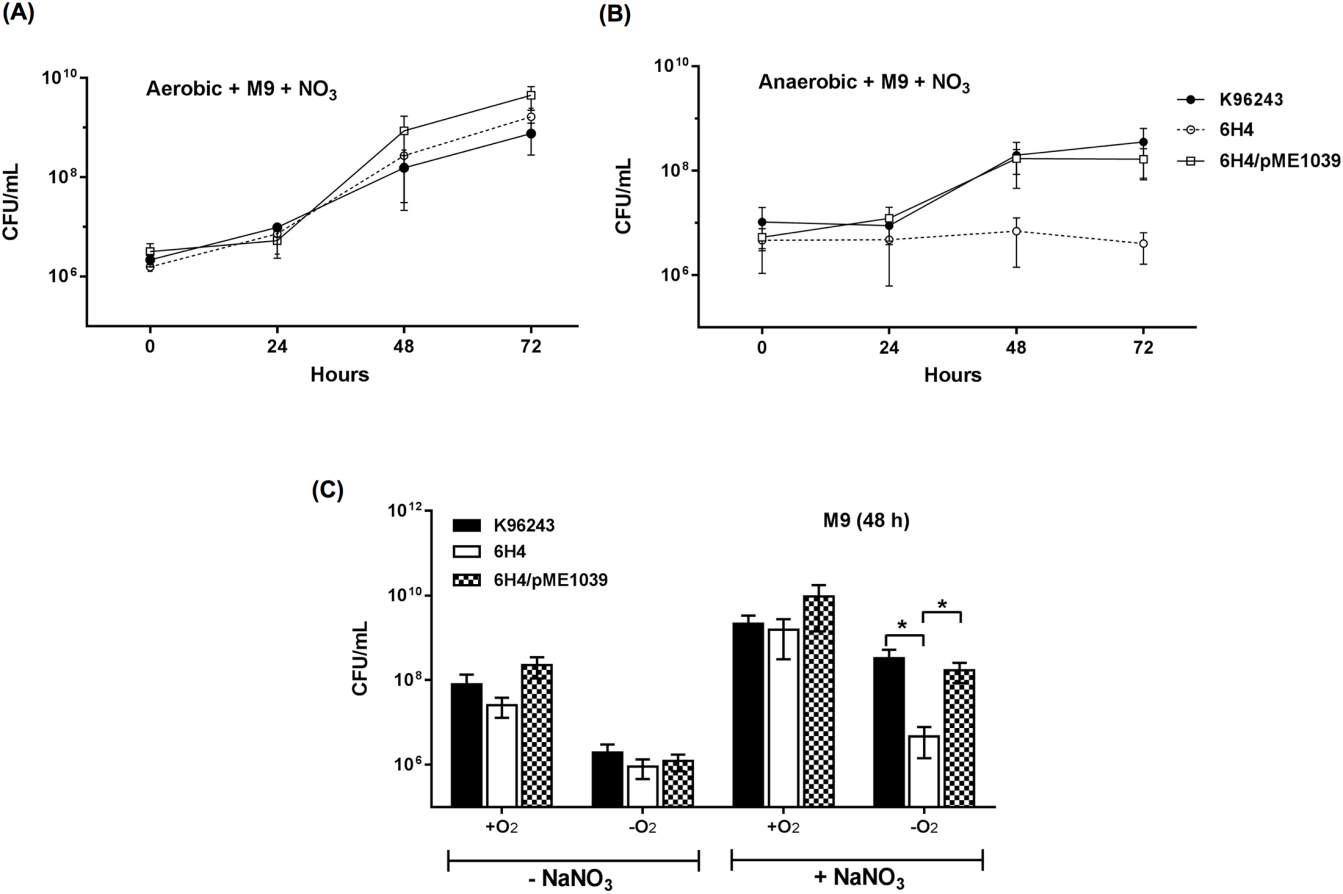
Effect of nitrate and anaerobic culture conditions on *B*. *pseudomallei* growth. *B*. *pseudomallei* was inoculated in M9 minimal medium supplemented with (+NaNO_3_) or without (-NaNO_3_) sodium nitrate, and incubated under aerobic or anaerobic culture conditions. (A) anaerobic and (B) aerobic kinetic growth curves of *B*. *pseudomallei* cultured in the presence of nitrate. *B*. *pseudomallei* K96243 wild-type (black circle), 6H4 mutant (white circle), 6H4/pME1039 complementation (white square) strains were grown for 72 h. Every 24 h, the numbers of viable bacteria were determined by plating on LB agar for colony count. (C) Growths of *B*. *pseudomallei* K96243 wild-type (black bar), 6H4 mutant (white bar) and 6H4/pME1039 (checked bar) strains under aerobic (+O_2_) and anaerobic (-O_2_) culture conditions at 48 h after bacterial inoculation. Results are presented from at least three replicates with three independent experiments. Asterisks indicate statistically significant differences (*P* < 0.05, *t*-test).

To further investigate the function of BPSL1039-1040, we then investigated the growth of the *B*. *pseudomallei* 6H4 mutant and the wild-type strain in M9 minimal medium supplemented with sodium nitrate under anaerobic compared with aerobic conditions, as measured by bacterial colony count. We observed that the growth curves of *B*. *pseudomallei* 6H4 in M9 medium supplemented with sodium nitrate were not significantly different in aerobic conditions (Fig 3A). However, there was a significantly reduced amount of growth compared with the wild-type strain under anaerobic conditions (Fig 3B). In addition, there was no significant difference in the growth of *B*. *pseudomallei* wild-type versus 6H4 mutant strains under aerobic conditions, whether the medium was supplemented with sodium nitrate or not (Fig 3C). This indicated that BPSL1039-1040 was active under anaerobic conditions, and that it may be a nitrate importer for bacterial metabolism and growth. Complementation with plasmid-borne *bpsl1039* could restore the growth defect to that of the wild-type level (Fig 3B and 3C). Accordingly, a single *bpsl1039* gene is able to complement bacterial growth defect of 6H4 mutant.

### *B*. *pseudomallei* 6H4 mutant showed decreased biofilm formation only under anaerobic conditions

**Fig 4:**
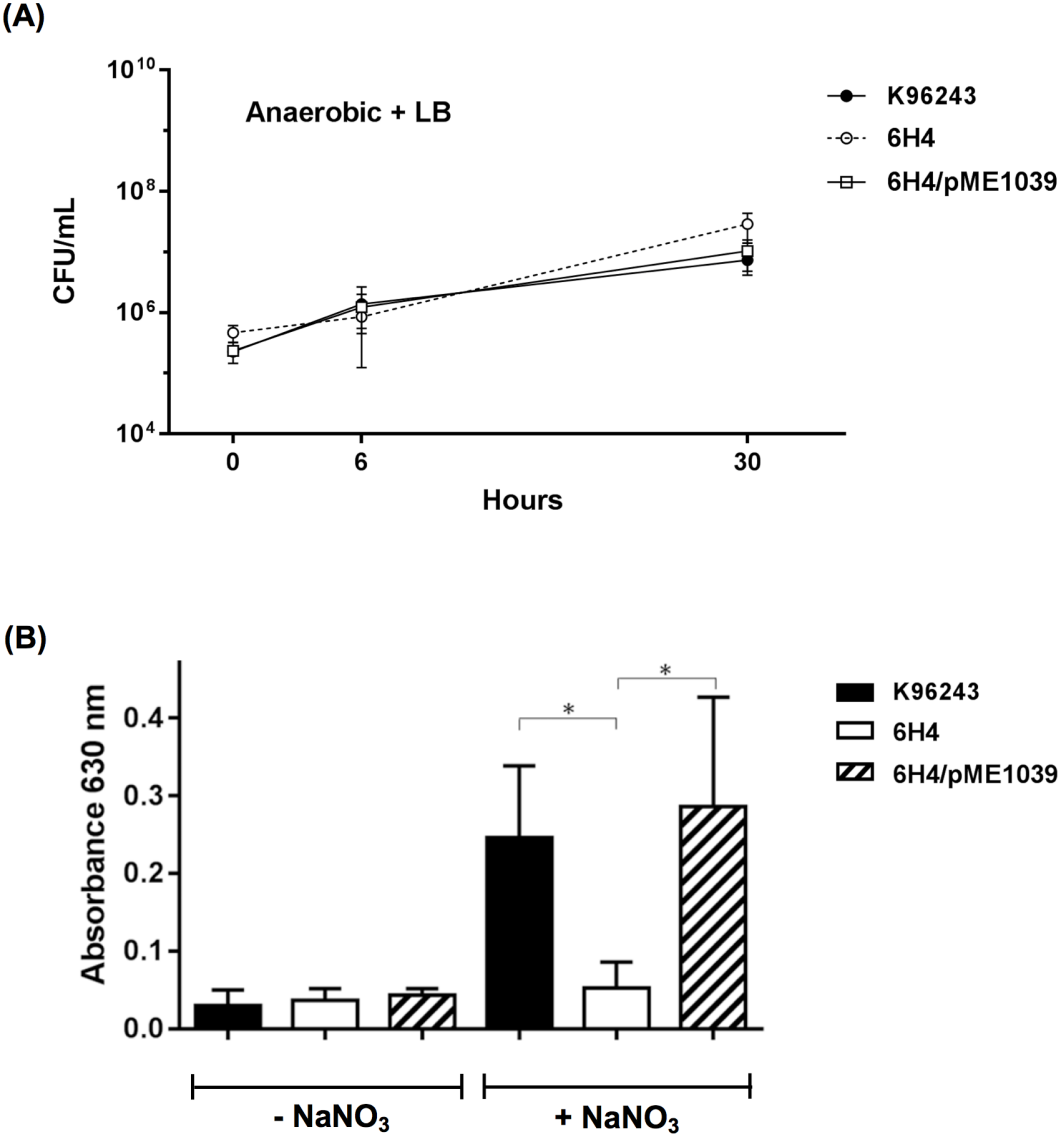
Biofilm formation of *B*. *pseudomallei* wild-type and its derivative strains. (A) Anaerobic kinetic growth curves of *B*. *pseudomallei* cultured in LB medium. *B*. *pseudomallei* wild-type K96243 (black circle), *bpsl1039 Tn* mutant (white circle), *bpsl1039*/pME1039 (square) strains were grown, and the numbers of viable bacteria were determined by plating on LB agar to obtain colony forming units (CFU). (B) *B*. *pseudomallei* K96243, *bpsl1039 Tn* mutant and *bpsl1039*/pME1039 strains were cultured in LB medium without (-NaNO_3_) or with (+NaNO_3_) sodium nitrate supplementation under aerobic (+O_2_) or anaerobic (-O_2_) conditions. A 96-well plate was incubated for three days. The degree of biofilm formation of *B*. *pseudomallei* K96243 wild-type (black bar), 6H4 (white bar) and 6H4/pME1039 (striped bar) strains were measured using crystal violet stain at OD630 nm. Three biological replicates were used, each with eight technical repeats. Results are presented as standard errors of the means for experiments done in triplicate, with five independent experiments. Asterisks indicate significant differences (*P* < 0.05, *t*-test).

Biofilm is a community of microorganisms that attach to a surface embedded in an extracellular matrix, and it is induced by a variety of environmental stresses, such as low oxygen [26]. Therefore, biofilm formation of *B*. *pseudomallei* wild-type and 6H4 mutant were analysed in LB broth with and without nitrate supplementation under aerobic and anaerobic conditions. The reason for testing biofilm formation in an LB medium instead of an M9 medium is to avoid the growth effect (Fig 4A). This revealed that in the absence of nitrate supplementation, no significant difference could be observed in biofilm formation between *B*. *pseudomallei* wild-type and the mutant derivative under all conditions tested (Fig 4B). However, when nitrate was included in the LB medium, the biofilm-forming efficiency of the 6H4 mutant under anaerobic conditions was significantly reduced compared with the wild-type strain (Fig 4B). This indicated that the presence of nitrate could enhance biofilm formation only under anaerobic conditions. This finding is supported by a previous study showing that the facultative anaerobe *B*. *pseudomallei* is likely to use nitrate for anaerobic growth in a biofilm [27]. Complementation of the 6H4 mutant with the plasmid-borne *bpsl1039* gene was able to restore biofilm formation to the same extent as the wild-type level (Fig 4B).

Although there is no general growth defect of the 6H4 mutant in LB broth under anaerobic culture conditions (Fig 4A), one must consider that the environment within a biofilm could be different. Therefore, further studies are required to elucidate the exact mechanism to explain the reduced biofilm-forming capabilities of the 6H4 mutant.

*B*. *pseudomallei* is often found in deep, wet soil, a microenvironment that is known to have little or no oxygen [28]. During the course of human infection, this bacterium is likely to encounter low oxygen in different tissue types and organs. For example, abscesses have been reported to become completely devoid of oxygen [29]. The persistence of *B*. *pseudomallei* under oxygen-limited conditions is likely an important factor for its survival and pathogenesis in humans. It has been postulated that biofilm formation may promote bacterial survival or spreading within the host as well as acting as a matrix shield against the host immune system. Moreover, the biofilm-forming ability of *B*. *pseudomallei* is believed to be associated with cases of chronic and relapse melioidosis [30]. The reduced biofilm-forming capability of the 6H4 mutant under anaerobic conditions suggested that BPSL1039-1040 ABC transporter may be a contributing factor of *B*. *pseudomallei* pathogenesis, especially in an oxygen-depleted environment.

### *B*. *pseudomallei* 6H4 mutant exhibited decreased efficiency of plaque formation

There is increasing evidence that ABC systems play important roles in the virulence of bacteria, and they have been linked with the requirement for uptake and/or maintenance of essential nutrients. For example, the Vps/VacJ ABC transporter in *S*. *flexneri* involved in the maintenance of lipid in the bacterial outer membrane is required for intracellular spread [31]. Similarly, mutation of group B streptococcus *glnQ* (involved in glutamine transport) showed decreased adherence to, and invasion of, respiratory epithelial cells *in vitro*, and decreased virulence *in vivo* [32].

**Fig 5:**
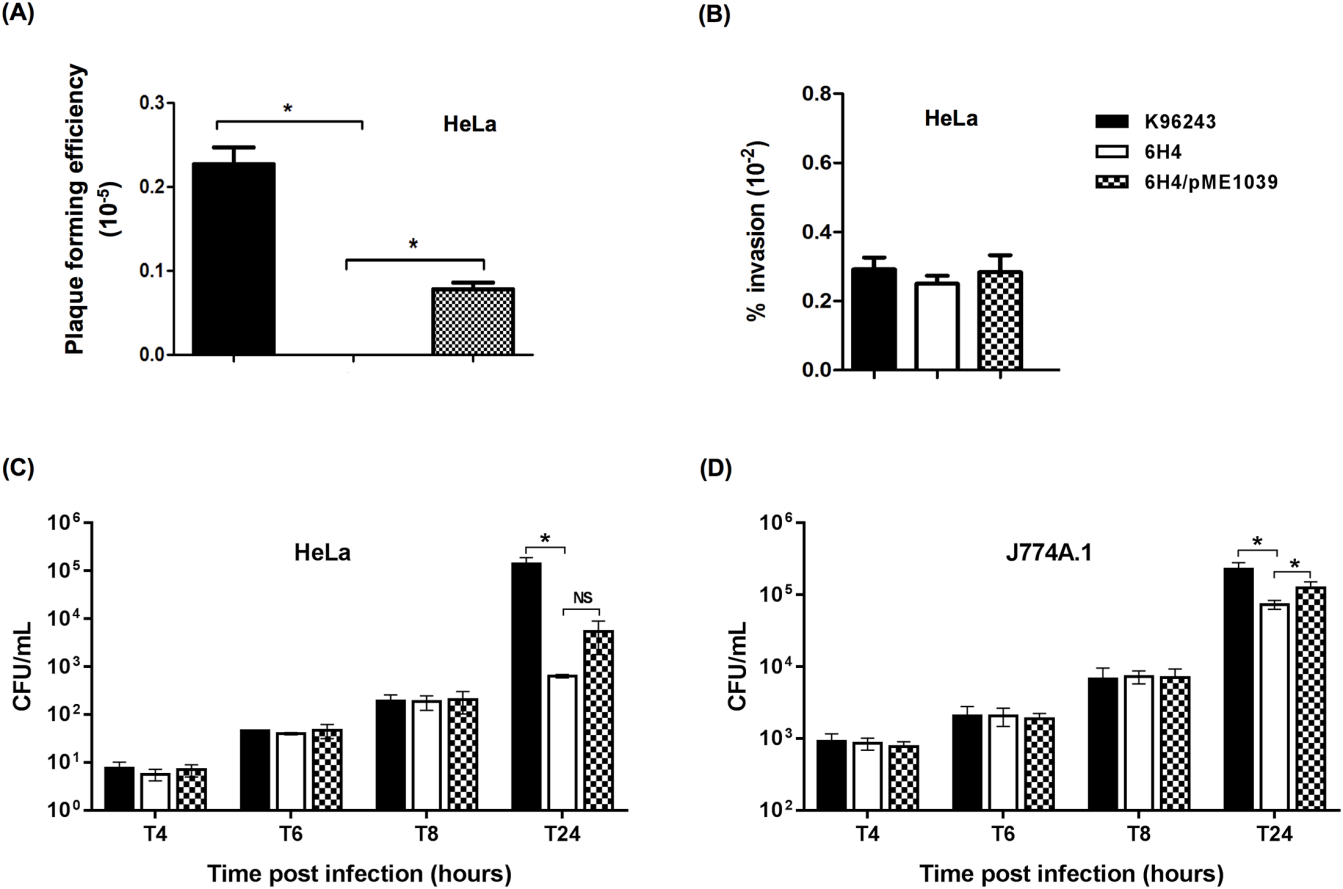
Plaque forming, invasion efficiencies and net intracellular survival and replication of *B*. *pseudomallei* wild-type and its derivative strains. (A) Plaque forming and (B) invasion efficiencies of *B*. *pseudomallei* K96243 wild-type, 6H4 mutant or 6H4/pME1039 complemented strains in infect HeLa cells. Plaque-forming efficiency was calculated as: number of plaques/bacterial CFU added per well. Percent invasion was determined as: (number of intracellular bacteria post infection/number of CFU added) × 100. *B*. *pseudomallei* K96243 wild-type (black bar), 6H4 mutant (white bar) and the 6H4/pME1039 complemented (checked bar) strains were used to infect (C) HeLa and (D) J774A.1 macrophage cells. Intracellular loads of bacteria were enumerated at 4, 6, 8, and 24 h p.i. Asterisks indicate significant differences (*P* < 0.05, *t*-test) between wild-type and its derivative strains. Results are presented as standard errors of the means for experiments done in triplicate, with three independent experiments.

Due to the possible attenuation of the 6H4 mutant, we further investigated the virulence of this mutant in a tissue culture model of infection. As shown in Fig 5A, *B*. *pseudomallei* 6H4 mutant exhibited significantly reduced plaque-forming efficiency in HeLa cells compared with the wild-type strain. An invasion assay revealed that the observed deficiency in plaque formation was not due to an inability of the mutants to enter the host cells because no significant differences in the invasion efficiencies of the wild-type and the 6H4 mutant to HeLa cells were observed (Fig 5B). Complementation by plasmid-borne *bpsl1039* could restore the plaque forming efficiency of the 6H4 mutant (Fig 5A).

### *B*. *pseudomallei* 6H4 mutant showed defective survival in infected phagocytic and non-phagocytic cells

To further investigate whether plaque formation results were linked to the inability of bacteria to replicate inside host cells, *B*. *pseudomallei* wild-type and 6H4 mutant were used to infect either HeLa cells or J774A.1 murine macrophages. This revealed that at 24 h p.i., the 6H4 mutant showed significantly reduced survival in HeLa cells and J774A.1 macrophages compared to the wild-type strain (Fig 5C and 5D). This result suggested that *B*. *pseudomallei* BPSL1039-1040 ABC transporter may be responsible for nitrate acquisition in infected cells and may promote the advantage to the bacteria. Our suggestion is supported by previous studies that ABC transporters are important in bacterial intracellular survival by transporting nutrients, such as the D-alanine transporter of *Salmonella enterica* [33] and the glutamine transporter of *Campylobacter jejuni* [34].

Complementation of the 6H4 mutant with plasmid-borne *bpsl1039* could restore this defect in J774A.1 cells. Although, the plasmid-borne *bpsl1039* gene could increase the number of viable intracellular *B*. *pseudomallei* 6H4/pME1039 complemented strain in HeLa cells at 24 h p.i. to 5,387 ± 2,082 CFU, compared with the 6H4 mutant (637 ± 31 CFU); however, this was not statistically different.

In this study, the phenotypes observed of the 6H4 complemented strain in cell cultures could be restored by introducing only *bpsl1039* gene. The expression of *bpsl1039* in the complemented strain was confirmed by RT-PCR analysis (data not shown). The possible explanation for the ability of *bpsl1039* to restore the *bpsl1039-1040* mutation effect may due to the ATPase substitution from another ABC transporter of *B*. *pseudomallei*. An analysis of the *B*. *pseudomallei* genome revealed a total of 338 ABC system-associated open reading frames [9]. It had been reported in *Streptococcus mutans*, where the ATPase function of different ABC transporters can be compensated by other ATPases encoded in other regions of the chromosome [35]. In addition, based on informatics analysis, a single ATPase protein can serve as several ABC transporters [36].

Many attempts to construct a 6H4 mutant complemented with both *bpsl1039-1040* genes expressed from a plasmid vector were carried out. Unfortunately, no complemented strain could be assembled. This failure to generate a double complementation may be due to difficulty in introducing a large recombinant plasmid (approximately 12 kb) that carried both *bpsl1039-1040* locus into *B*. *pseudomallei*, or the adverse effects from the high expression of either *bpsl1040* or both *bpsl1039* and *bpsl1040*.

### BPSL1039-1040 is essential for virulence of *B*. *pseudomallei* in mice

**Fig 6:**
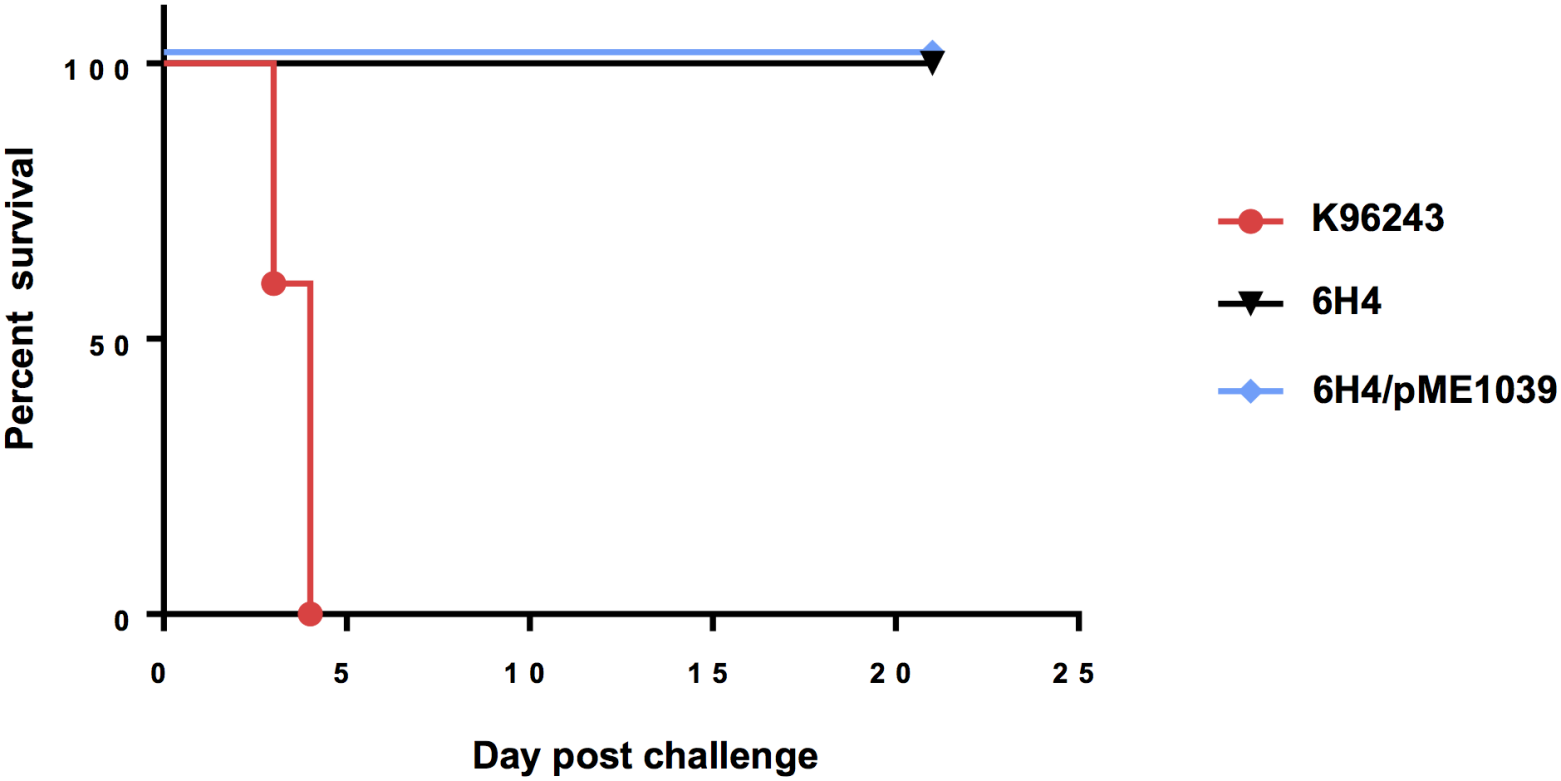
*B*. *pseudomallei bpsl1039* 6H4 mutant is attenuated in mice. Survival of BALB/c mice inoculated *via* the intranasal route with *B*. *pseudomallei* K96243 wild-type (●), 6H4 (▼) and 6H4/pME1039 complemented (◆) strains were determined (n = 5 per group). The survival curves of mice infected with wild-type and 6H4 were significantly different (*P* < 0.01). Data were analysed using the Log-rank (Mantel-Cox) test with a Bonferroni correction.

Anaerobic fitness of pathogens is often required for their pathogenesis. The impaired ability of the 6H4 mutant to acquire nitrate and its defective survival in the tissue culture model of infection might contribute to *B*. *pseudomallei* virulence in the animal model. As shown in Fig 6, mice challenged intranasally with *B*. *pseudomallei* K96243 wild-type died within 5 days of infection. The *B*. *pseudomallei* 6H4 mutant was attenuated, with a 100% survival rate in mice intranasally challenged with 1,700 CFU of the mutant. The 6H4 mutant complemented with plasmid-born *bpsl1039* showed an attenuation similar to the mutant strain. The inability of the *bpsl1039* gene alone to complement the attenuation of the 6H4 mutant in the mouse model, as opposed to the cell culture assays, is likely due to the lack of IPTG induction in the mouse model. The plasmid used for complementation contains *lacI*^*Q*^ and requires IPTG for induction.

Upon termination of the challenge experiment at day 21, lungs and spleens from the infected mice were homogenized and quantified by viable plate count to determine if any remaining *B*. *pseudomallei* could be found in these organs. A total of 1×10^4^ and 2.5×10^4^ CFU *B*. *pseudomallei* were found in the lungs of mice challenged intranasally with the 6H4 mutant and the mutant complemented with *bpsl1039*, respectively. No bacteria could be detected in the spleens. The results demonstrating the presence of the bacteria in the lungs but not in the spleens agree with the original STM study [10].

Detection of the *B*. *pseudomallei* 6H4 mutant in the lungs but not in spleens may be due to an inability of the bacteria to spread beyond the initial site of infection. It has been reported that oxygen levels of mammalian (human) tissues (ranging from 33.8–48.9 mmHg) is much lower than those in lung alveoli (110 mmHg) [37]. High oxygen levels in the alveoli may allow the 6H4 mutant, which is defective for anaerobic growth, to undergo aerobic respiration, whereas oxygen tension in splenic tissues is much lower [37]. The role of nitrate as an electron acceptor during anaerobic respiration [38, 39] may explain its requirement for growth in secondary organs beyond the lung. This observation is supported by previous reports on the important roles of nitrate respiration in the blooming of Gammaproteobacteria, such as *E*. *coli* or *Salmonella enterica* serovar Typhimurium, in inflammatory conditions [40, 41].

Previously, using STM, several mutants were tested as live attenuated vaccine candidates in *B*. *pseudomallei* [10]. However, most mutants exhibited mild attenuation and provided limited protection. For example, an *aroB* mutant was attenuated in BALB/c mice [10] and provided partial protection. In the present study, the *B*. *pseudomallei* 6H4 mutant showed attenuation in the mouse model, which was in agreement with our *in vitro* testing; this suggests that the 6H4 mutant may be a possible candidate for further development as a live attenuated vaccine for melioidosis. Since *B*. *pseudomallei* were detected only in the lungs of 6H4-infected mice, we propose that a combination of 6H4 mutagenesis along with the mutation of another gene would generate the observed severe attenuation of the *B*. *pseudomallei* mutant.

## Conclusions

We found that BPSL1039-1040 was involved in *B*. *pseudomallei* nitrate metabolism under anaerobic conditions. *In vitro* assays revealed that the *B*. *pseudomallei* 6H4 mutant had defects in plaque formation, survival in infected cells, and in biofilm formation. *In vivo* studies using an intranasal mouse model of infection demonstrated an attenuated virulence of the 6H4 mutant. The possible explanation may be due to a reduced ability to acquire nitrate from the environment, resulting in low bacterial burdens under anaerobic conditions.

## Supporting information

S1 Table Human-endpoints-checklist. (DOCX)

S1 Fig Three-dimensional structure of *B*. *pseudomallei* BPSL1039, an integral trans- membrane protein of ABC transporter. (A) Structural model of *B*. *pseudomallei* BPSL1039 transporter membrane protein. (B) Nitrate was docked into the BPSL1039 ABC transporter binding site. Interactions between nitrate and Thr 480 are shown as purple dotted lines. Nitrate is shown in ball-and-stick representation, and amino acid residues located in binding site are shown in line mode. Figures were generated with Discovery Studio Visualizer-Accelrys.
(TIFF)
