# Supplementary material for: Inactivation of bpsl1039-1040 ATP-binding cassette transporter reduces intracellular survival in macrophages, biofilm formation and virulence in the murine model of Burkholderia pseudomallei infection
Source: PLoS One. 2018 May 17;13(5):e0196202. doi: 10.1371/journal.pone.0196202 (PMC5957425; doi:10.1371/journal.pone.0196202)
Supplement: S1 Table — (DOCX) [file pone.0196202.s001.docx]

*PLOS ONE* Humane Endpoints Checklist

*PLOS ONE* manuscript number: ____ **PONE-D-17-19057 - [EMID:6201900c771fbcae]**

Title: Inactivation of *bpsl1039-1040* ATP-Binding Cassette Transporter Reduces Intracellular Survival in

Macrophages, Biofilm Formation and Virulence in the Murine Model of *Burkholderia pseudomallei*

Infection

**Complete the following if your study design includes death of a regulated animal as a likely outcome or planned experimental endpoint. Please also include all information in the Methods section of your manuscript.**

**ITEM 1.** **Describe whether humane endpoints* were used for all animals involved in the study.**

|  | **Recommendation** | **Section/Paragraph** |
| --- | --- | --- |
| **If humane endpoints* were used, report the following:** | | |
| **1** | **The specific criteria used to determine when animals should be euthanized** | The mice were routinely checked twice daily for signs of illness. If it was determined that they were likely to reach the humane endpoint specified in the Project Licence before the next monitoring event (weight loss exceeding 20% plus signs of clinical illness scored numerically, including an assessment of piloerection, hunching, movement, respiratory pattern, and grimace scale), the mice were immediately culled by cervical dislocation.  **(Materials and Methods section; p.10, lines 224-8)** |
| **2** | **Once animals reached endpoint criteria, the amount of time elapsed before euthanasia** | **Once mice reached endpoint criteria, they were immediately culled.**  **(Materials and Methods section; p.10, line 228)** |
| **3** | **Whether any animals died before meeting criteria for euthanasia** | **none** |
| **If humane endpoints* were not used, report the following:** | | |
| **1** | **A scientific and ethical justification for the study design, including the reasons why humane endpoints could not be used, and discussion of alternatives that were considered but could not be used** |  |
| **2** | **Whether the institutional animal ethics committee specifically reviewed and approved the anticipated mortality in the study design** |  |

**ITEM 2.** **Include the following details of the study design and outcomes.**

|  | **Recommendation** | **Section/Paragraph** |
| --- | --- | --- |
| **1** | **The duration of the experiment** | **14-30 days.**  **(Materials and Methods section; p.10, line 223)** |
| **2** | **The numbers of animals used, euthanized, and found dead (if any); the cause of death for all animals** | **25 animals were used, of which 10 were culled at end of experiment and 15 culled in anticipation of reaching the humane end point. Euthanasia was cause of death for all animals.**  **None found dead.**  **(Materials and Methods section, p.11, lines 233-5),** |
| **3** | **How frequently animal health and behavior were monitored** | **Twice daily.**  **(Materials and Methods section; p.10, line 224)** |
| **4** | **All animal welfare considerations taken, including efforts to minimize suffering and distress, use of analgesics or anaesthetics, or special housing conditions** | The mice were housed under specific pathogen-free conditions, with free access to food and water and environmental enrichment (including Nestlets).  **(Materials and Methods section; p.10, lines 211-3)** |
| **5** | **Any special training in animal care or handling provided for research staff** | **All the animal care workers have over 15 years of experience working with laboratory animals including B. pseudomallei-infected mice. They also hold qualifications as NACWO or NTCO.**  **(Materials and Methods section; p.11, lines 235-8)** |

***Definition of a humane endpoint**

A humane endpoint is an experimental endpoint at which animals are euthanized when they display early markers associated with death or poor prognosis of quality of life, or specific signs of severe suffering or distress. Humane endpoints are used as an alternative to allowing such conditions to continue or progress to death following the experimental intervention (“death as an endpoint”), or only euthanizing animals at the end of an experiment. Before a study begins, researchers define the practical observations or measurements that will be used during the study to recognize a humane endpoint, based on anticipated clinical, physiological, and behavioral signs. These may include, for instance, body temperature or weight changes, tumor size or appearance, abnormal behaviors, pathological changes, ruffled fur, reduced mobility, body posture, or expression of specific body fluid markers. Please see the NC3Rs guidelines for more information.

**ARRIVE Guidelines**

*PLOS ONE* encourages authors to follow the [Animal Research: Reporting of In Vivo Experiments (ARRIVE) guidelines](http://www.nc3rs.org.uk/arrive-guidelines) for all submissions describing laboratory-based animal research and to upload a completed [ARRIVE Guidelines Checklist](http://www.nc3rs.org.uk/sites/default/files/documents/Guidelines/NC3Rs%20ARRIVE%20Guidelines%20Checklist%20%28fillable%29.pdf) to be published as supporting information.
