# Supplementary figures and images for: Inactivation of bpsl1039-1040 ATP-binding cassette transporter reduces intracellular survival in macrophages, biofilm formation and virulence in the murine model of Burkholderia pseudomallei infection
Source: PLoS One. 2018 May 17;13(5):e0196202. doi: 10.1371/journal.pone.0196202 (PMC5957425; doi:10.1371/journal.pone.0196202)

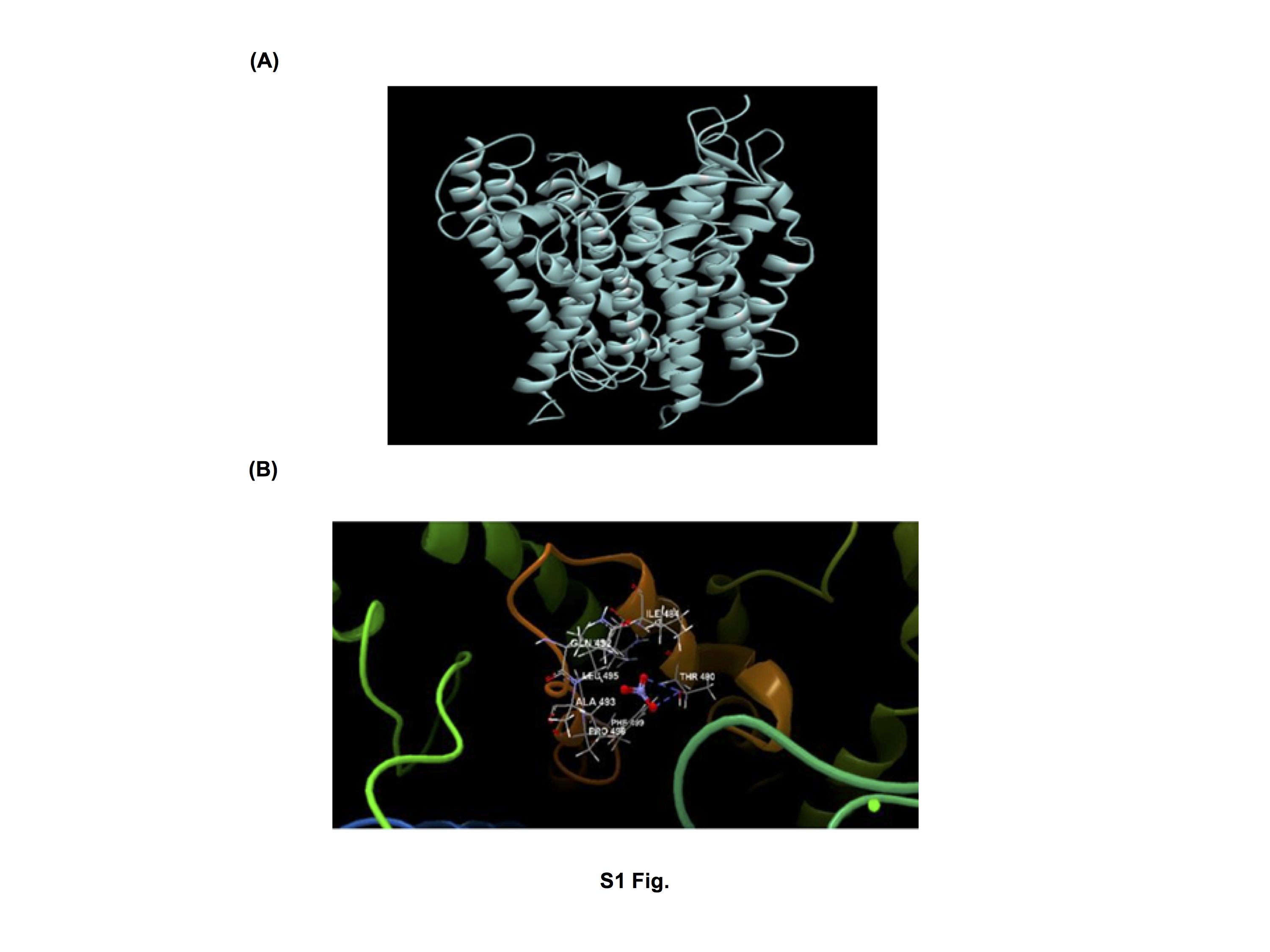

Supplement: S1 Fig — (A) Structural model of B. pseudomallei BPSL1039 transporter membrane protein. (B) Nitrate was docked into the BPSL1039 ABC transporter binding site. Interactions between nitrate and Thr 480 are shown as purple dotted lines. Nitrate is shown in ball-and-stick representation, and amino acid residues located in binding site are shown in line mode. Figures were generated with Discovery Studio Visualizer-Accelrys. (TIFF) [file pone.0196202.s002.tiff]
